# Supplementary material for: HisCoM-G×E: Hierarchical Structural Component Analysis of Gene-Based Gene–Environment Interactions
Source: Int J Mol Sci. 2020 Sep 14;21(18):6724. doi: 10.3390/ijms21186724 (PMC7555026; doi:10.3390/ijms21186724)
Supplement: Supplementary file 1 [file ijms-21-06724-s001.pdf]

Supplementary Materials

HisCoM-G×E: Hierarchical Structural Component Analysis of Gene-Based Gene–Environment Interactions

Sungkyoung Choi [1], Sungyoung Lee [2], Iksoo Huh [3], Heungsun Hwang [4] and Taesung Park [5,6,*]

[1] Department of Applied Mathematics, Hanyang University (ERICA), Ansan 15588, Korea; day0413@hanyang.ac.kr
[2] Center for Precision Medicine, Seoul National University Hospital, Seoul 03080, Korea; biznok@snu.ac.kr
[3] Department of nursing, College of Nursing and Research Institute of Nursing Science, Seoul National University, Seoul 03080, Korea; huhixoo@gmail.com
[4] Department of Psychology, McGill University, Montreal, Quebec H3A 1G1, Canada; heungsun.hwang@mcgill.ca
[5] Department of Statistics, Seoul National University, Seoul 08826, Korea; tspark@stats.snu.ac.kr
[6] Interdisciplinary Program in Bioinformatics, Seoul National University, Seoul 08826, Korea; tspark@stats.snu.ac.kr
* Correspondence: tspark@stats.snu.ac.kr; Tel.: +82-02-880-8924

(a) iSKAT

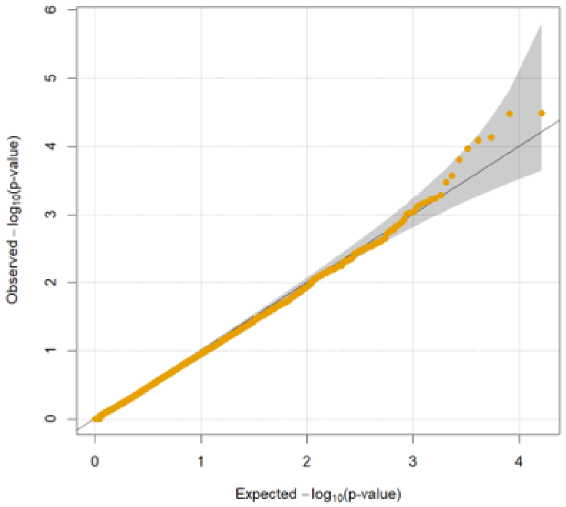

(b) GE_GATES

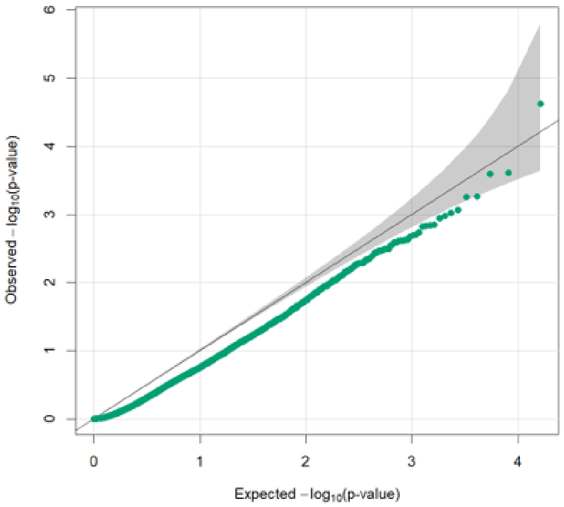

(c) HisCoM-G×E

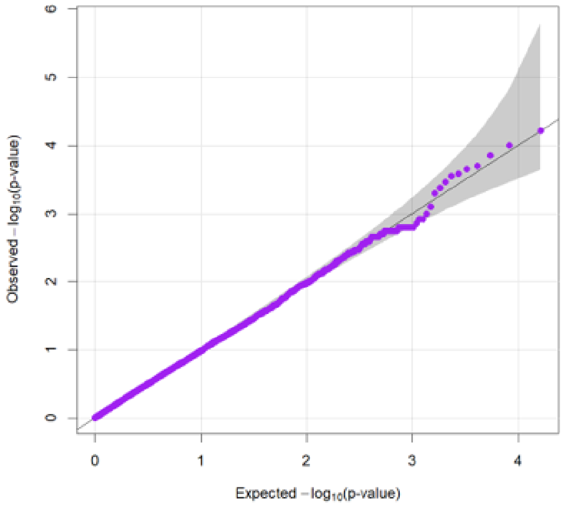

Figure S1. Quantile–quantile (QQ) plots of the G×E analysis for systolic blood pressure (SBP). QQ plots between observed and expected *p*-values for (**a**) iSKAT, (**b**) GE_GATES, and (**c**) HisCoM-G×E. The grey shading indicates a 95% confidence interval.

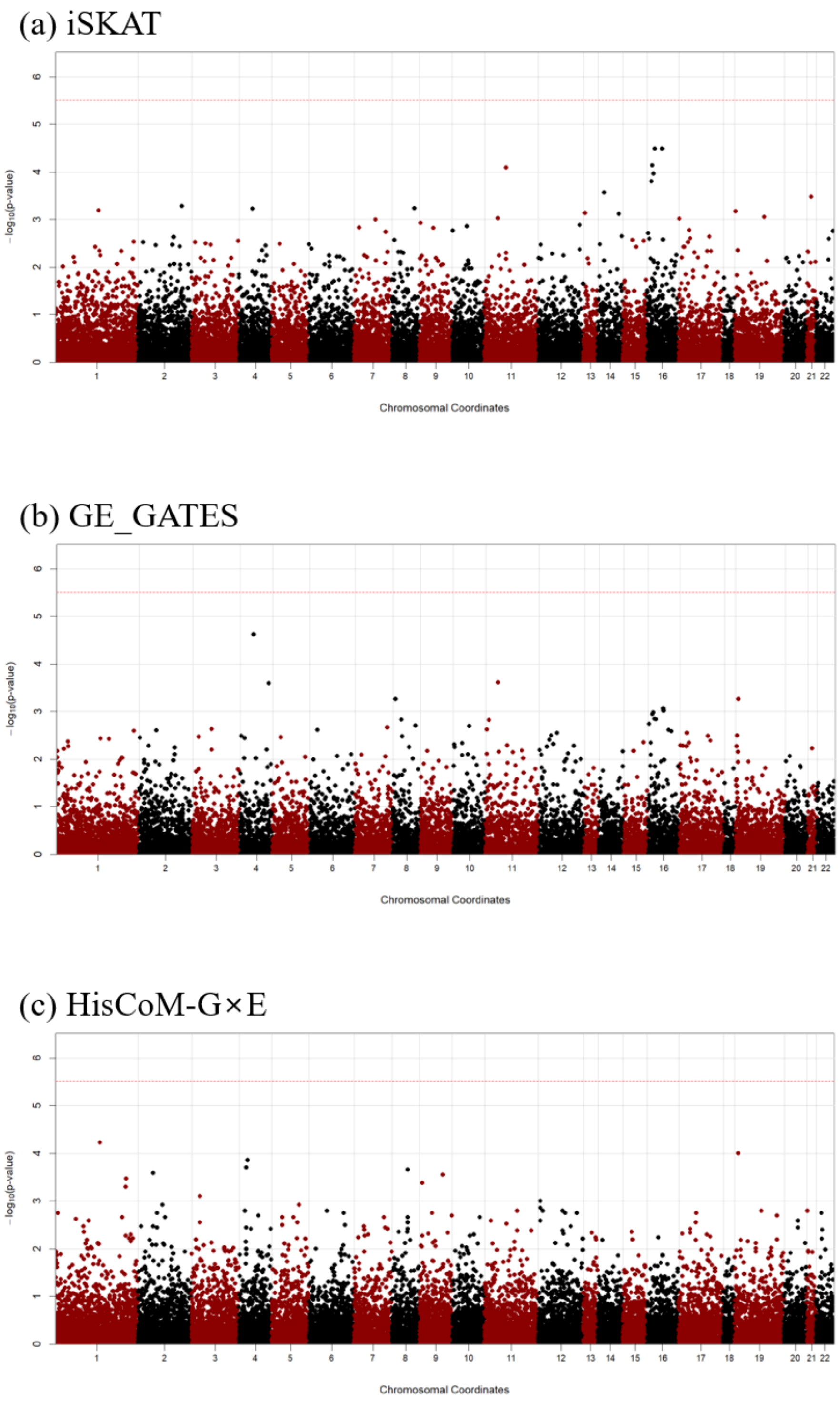

Figure S2. Manhattan plots of the G×E analysis for SBP. The Manhattan plots are provided for (**a**) iSKAT, (**b**) GE_GATES, and (**c**) HisCoM-G×E. The *x*-axis indicates the genome in physical position and the *y*-axis shows the $-\log_{10}(p\text{-value})$ for all genes. The red horizontal dashed line represents the threshold value of 3.06×10^{-6} for the 5% genome-wide significance level by Bonferroni correction.

Table S1. Empirical type I error estimates calculated, with 1000 replicates, at 0.05, 0.01, and 0.005 significance levels (α). Gene size was 5, 50, and 100 SNPs in a gene. The numbers of sample size are denoted by N.

Gene Size	N	α	GESAT	iSKAT	GE_minP	GE_GATES	GE_tTS	GE_tProd	HisCoM-G×E
5	500	0.05	0.050	0.046	0.052	0.065	0.050	0.051	0.041
		0.01	0.008	0.005	0.008	0.013	0.007	0.008	0.004
		0.005	0.003	0.003	0.005	0.006	0.003	0.002	0.002
	1000	0.05	0.049	0.061	0.053	0.064	0.049	0.049	0.063
		0.01	0.012	0.013	0.010	0.013	0.013	0.014	0.009
		0.005	0.007	0.003	0.006	0.009	0.006	0.008	0.006
	2000	0.05	0.053	0.057	0.037	0.058	0.054	0.052	0.071
		0.01	0.005	0.010	0.008	0.008	0.012	0.006	0.011
		0.005	0.003	0.006	0.002	0.005	0.007	0.004	0.004
50	500	0.05	0.048	0.045	0.000	0.042	0.046	0.046	0.058
		0.01	0.012	0.009	0.000	0.009	0.009	0.010	0.012
		0.005	0.007	0.004	0.000	0.005	0.003	0.004	0.005
	1000	0.05	0.046	0.055	0.000	0.025	0.045	0.044	0.040
		0.01	0.006	0.007	0.000	0.003	0.007	0.007	0.006
		0.005	0.002	0.001	0.000	0.002	0.003	0.002	0.002
	2000	0.05	0.060	0.057	0.000	0.042	0.052	0.051	0.049
		0.01	0.012	0.015	0.000	0.005	0.010	0.012	0.005
		0.005	0.007	0.007	0.000	0.000	0.004	0.006	0.002
100	500	0.05	0.056	0.042	0.000	0.018	0.055	0.053	0.044
		0.01	0.014	0.010	0.000	0.002	0.011	0.008	0.004
		0.005	0.006	0.002	0.000	0.000	0.005	0.005	0.002
	1000	0.05	0.057	0.058	0.000	0.020	0.060	0.059	0.053
		0.01	0.009	0.011	0.000	0.002	0.011	0.010	0.009
		0.005	0.00	0.008	0.000	0.000	0.008	0.007	0.005
	2000	0.05	0.055	0.049	0.000	0.024	0.055	0.056	0.045
		0.01	0.011	0.011	0.000	0.001	0.015	0.015	0.006
		0.005	0.005	0.005	0.000	0.001	0.001	0.008	0.002
